# Supplementary material for: Active colloidal molecules assembled via selective and directional bonds
Source: Nat Commun. 2020 May 29;11:2670. doi: 10.1038/s41467-020-16506-z (PMC7260206; doi:10.1038/s41467-020-16506-z)
Supplement: Supplementary file 1 — Supplementary Information [file 41467_2020_16506_MOESM1_ESM.pdf]

Supplementary Information

Active Colloidal Molecules Assembled via Selective and Directional Bonds

Wang et al.

Contents:

Supplementary Table 1 Page 2

Supplementary Figure 1-20 Page 3-24

Supplementary Discussion 1-4 Page 20-26

Supplementary References Page 26

Supplementary Tables.

Supplementary Table 1. List of metallodielectric patchy particles and the corresponding sizes.*

	P_I	P_{II} (P_1)	P_{III}	P_{IV}	P_V	P_{VI}(P_3)	P_{VII}	P_{VIII}(P_4)	P_{IX} (P_2)
Gold lobe dimeter (d_{gold}) (μm)	1.63	1.63	1.63	1.63	1.63	1.63	1.63	1.63	1.46
Dielectric lobe dimeter (d_{sphere}) (μm)	1.26	1.34	1.41	1.45	1.59	1.60	1.61	1.77	1.17
$d_{dielectric\ lobe}/D_{central\ sphere}$	0.3162	0.3369	0.3520	0.3631	0.3969	0.4015	0.4174	0.4421	0.2925

*The SEM images of these particles are shown in **Supplementary Figure 6**.

Supplementary Figures.

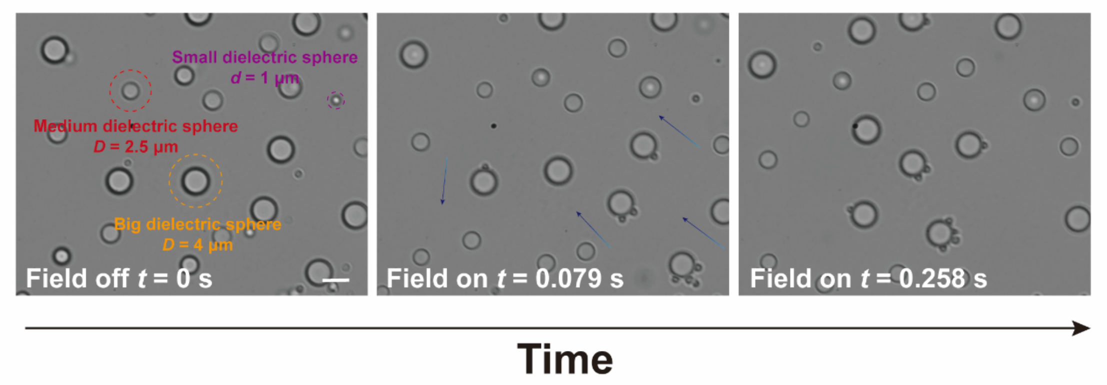

Supplementary Figure 1. Size selectivity. Colloidal molecules assembled by mixing dielectric spheres 1-, 2.5- and 4-µm in diameter. The 4-µm particle selectively assembles with the 1-µm particle, while the 2.5-µm particle shows no assembly with either 4-µm or 1-µm particles. Scale bar: 4 µm.

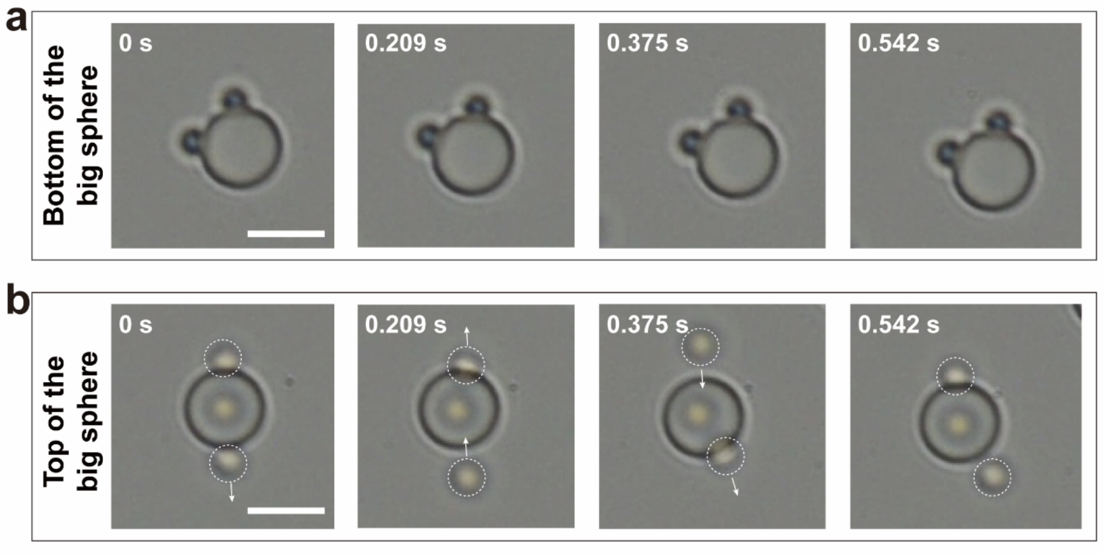

Supplementary Figure 2. Tracer particles surrounding a central dielectric sphere (4 μm) indicate the direction of EHD flow. Close to the substrate, the tracer particles (PS spheres, 1 μm) form stable bonds with the central sphere (**a**) while they are ejected away at the top (**b**). This indicates an inward EHD flow with respect to the central sphere close to the substrate. Scale bar: 4 μm.

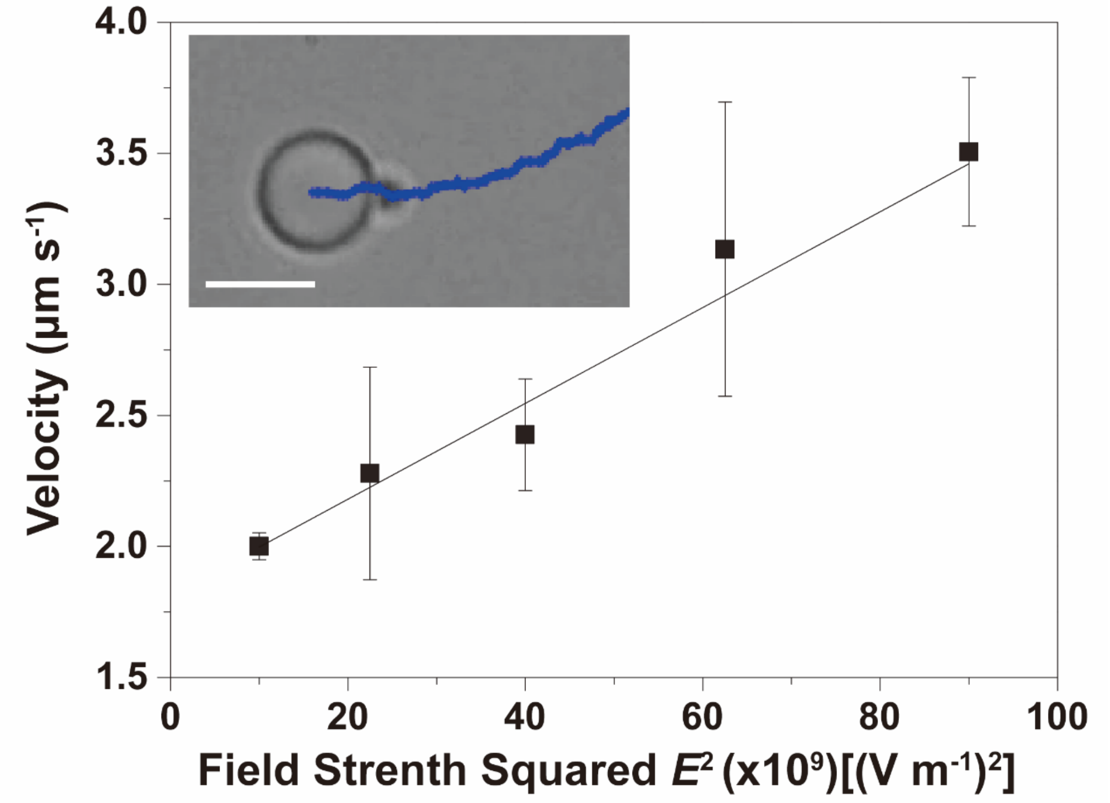

Supplementary Figure 3. The v~E^2 plot of AB colloidal molecules assembled by 4-μm central dielectric sphere and 1-μm ligand spheres. The velocity v of colloidal molecules (black square) scales linearly to the field strength squared E^2. The error bars refer to the standard deviation of velocities for multiple experiments. Inset shows an AB colloidal molecule with its propulsion trajectory.

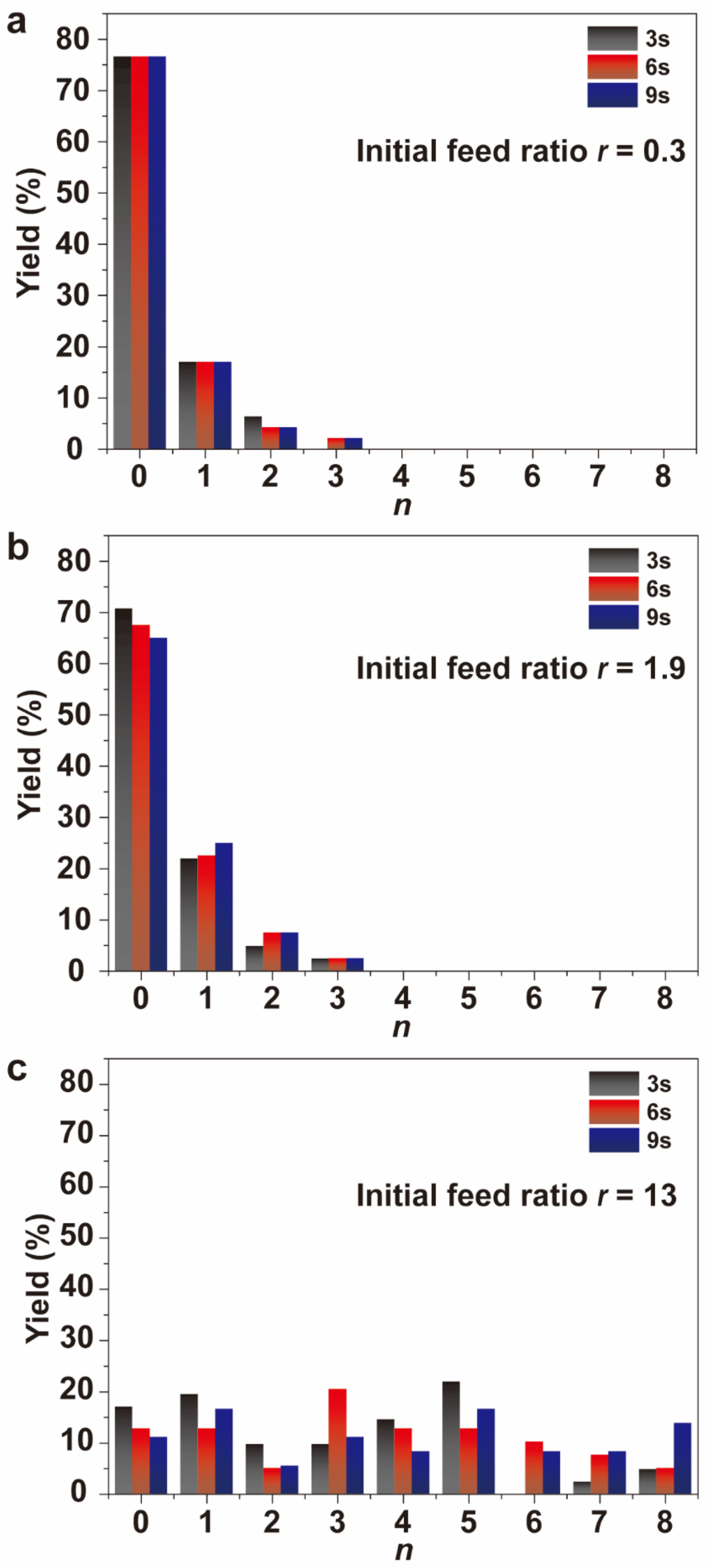

Supplementary Figure 4. Statistics of the yield of AB_n colloidal molecules. The yield of high-order colloidal molecules can be varied by changing the initial feed ratio of the ligand particles to the central dielectric spheres, as well as by extending the assembly time. Data for r=0.3 (**a**), 1.9 (**b**), and 13 (**c**) are collected at 3, 6, and 9 seconds of assembly, as labeled.

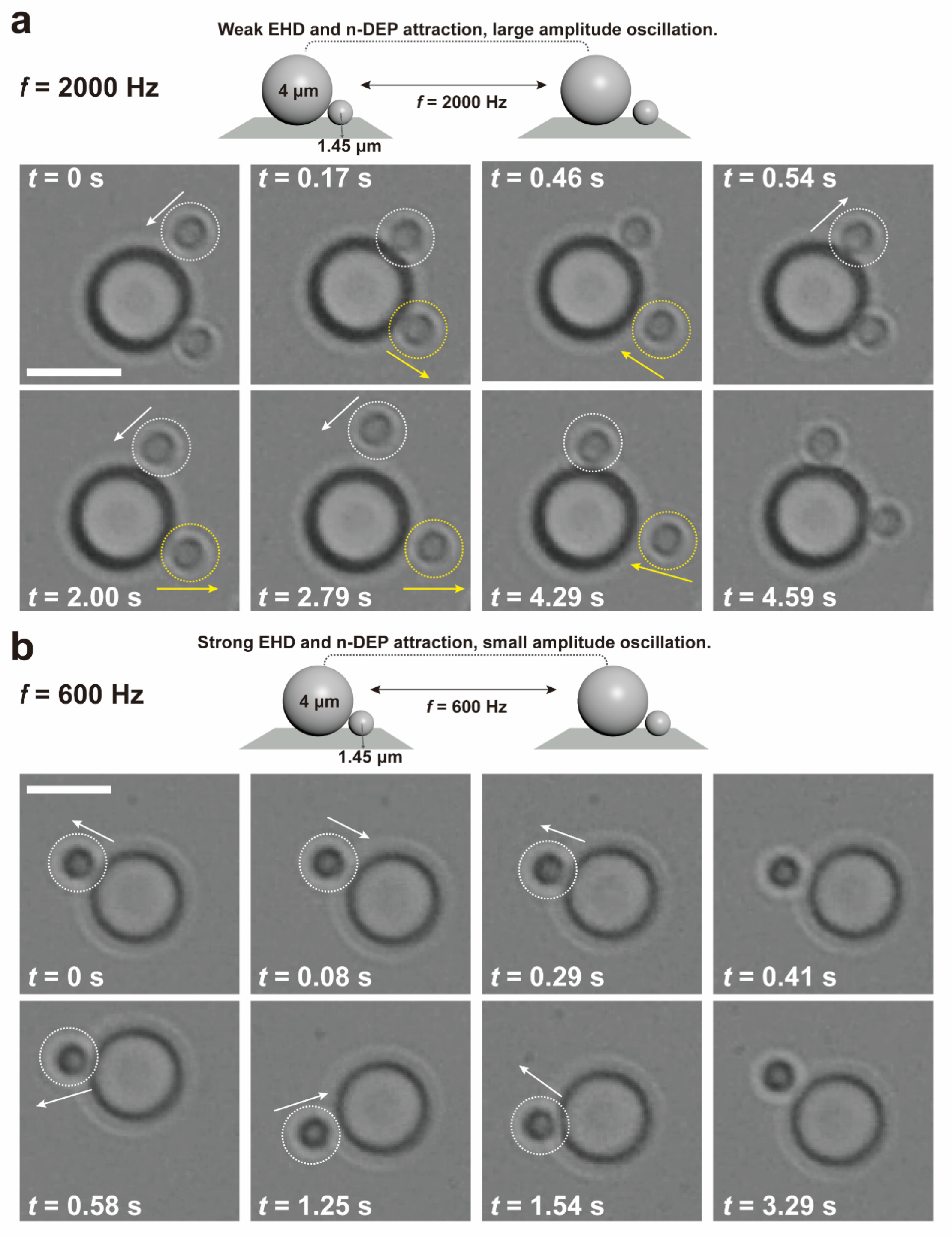

Supplementary Figure 5. Oscillation between the 4-μm and 1.45-μm dielectric sphere showing the critical size ratio for bonding. The 1.45-μm dielectric sphere shows oscillation (bonding and un-bonding) when assembling with the big sphere (D = 4 μm). The 1.45-μm sphere shows a large amplitude of oscillation at 2,000 Hz (**a**) compared to that at 600 Hz (**b**) due to the stronger attraction provided by n-DEP force and EHD force. Scale bar: 4 μm.

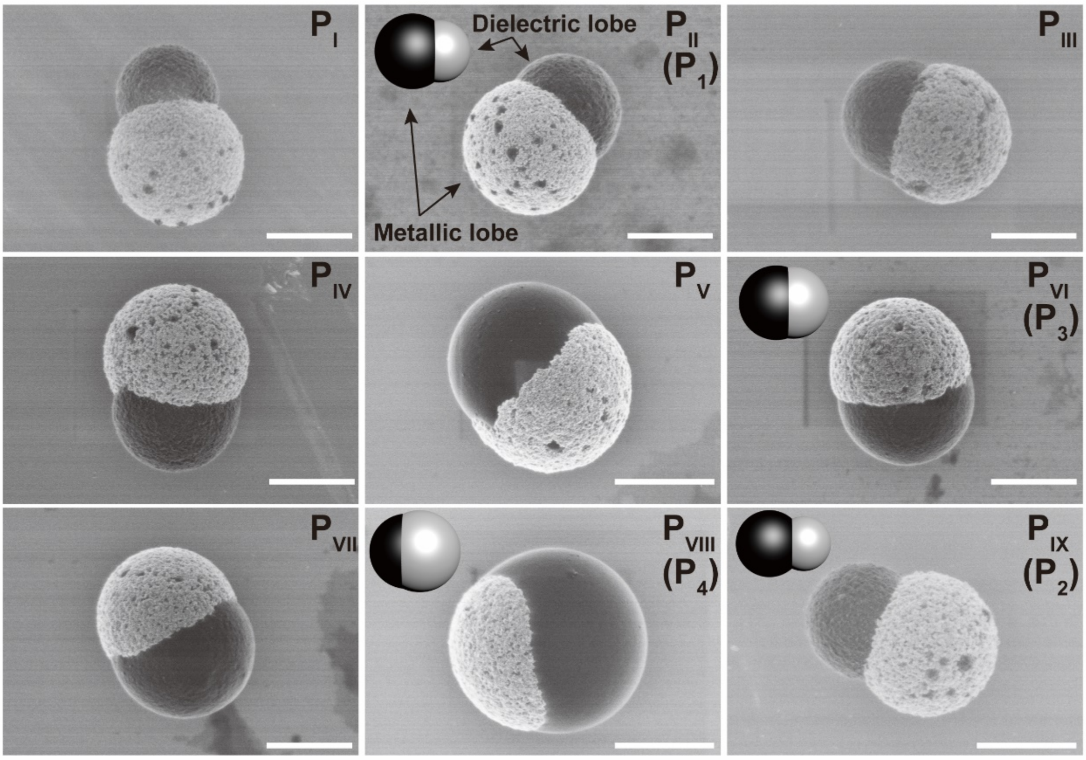

Supplementary Figure 6. Scanning electron microscope (SEM) images of metallodielectric patchy particles used for the assembly of colloidal molecules. The sizes of metallic (gold) lobes are constant while the sizes of the dielectric lobes increase from P_I to P_{VIII}. Both the dielectric and metallic lobes of P_{IX} are smaller than other particles. Insets are cartons of patchy particles $\mathbf{P_1}$ to $\mathbf{P_4}$, which are discussed in the main text; they are drawn strictly to the scale reflecting the shapes. In the SEM images, the metallic lobe appears bright while the dielectric lobe appears dark. This is opposite for optical microscope images of the same particles. The cartoons are made according to the optical microscope images and thus show contrast to the SEM images. The two parts are labeled with arrows to further illustrate the particle composition. Scale bar: 1 μm.

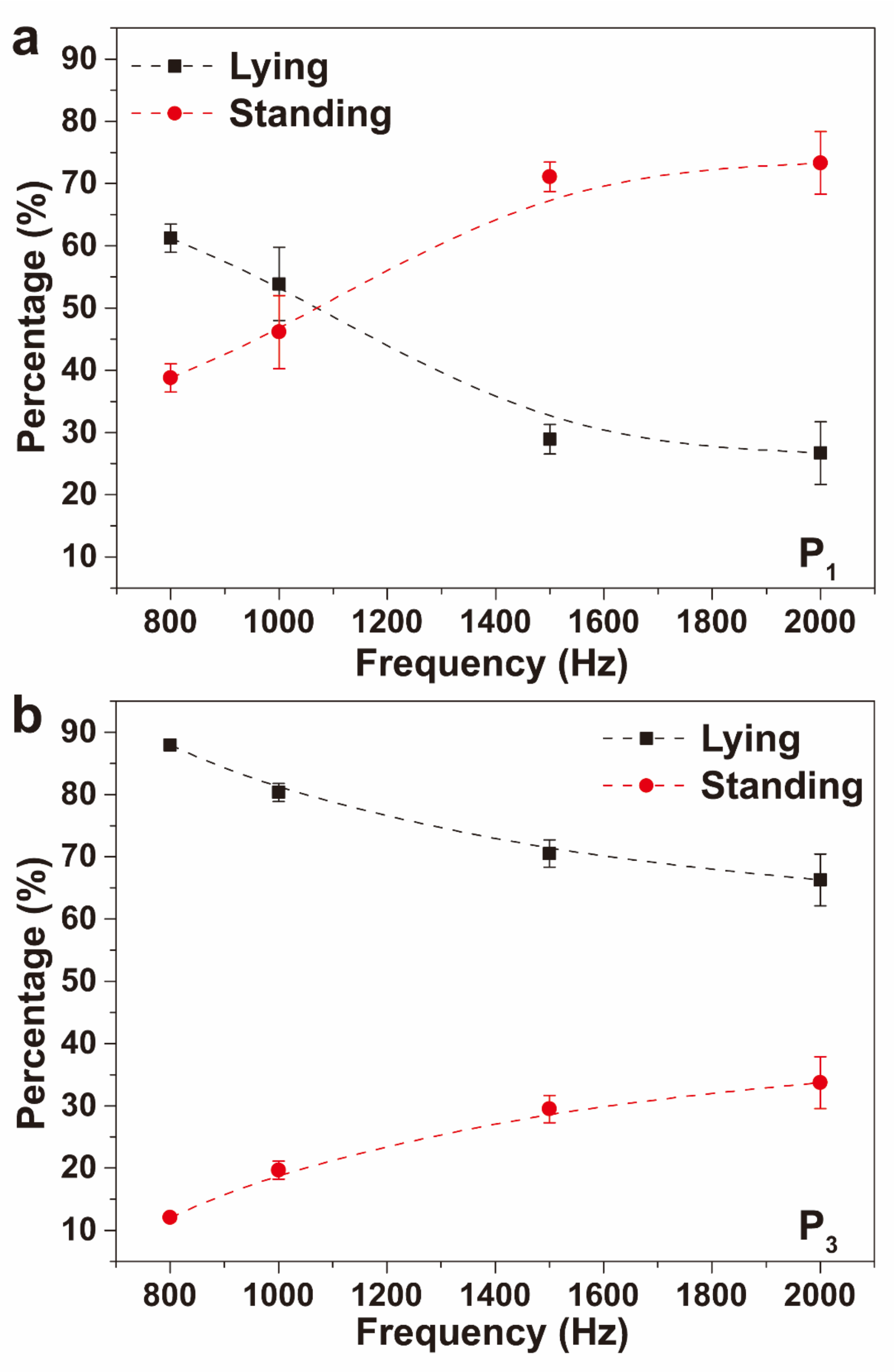

Supplementary Figure 7. Statistics of standing (static) and lying (propelling) patchy particle P_1 and P_3. The population of lying (black square) and stand-up particles (red dot) of P_1 (**a**) and P_3 (**b**) are recorded. Patchy particles may stand up under electric field to be aligned to the field. Only those lie on the substrate are able to propel by an ICEP mechanism. The error bars refer to the standard deviation of values for multiple experiments.

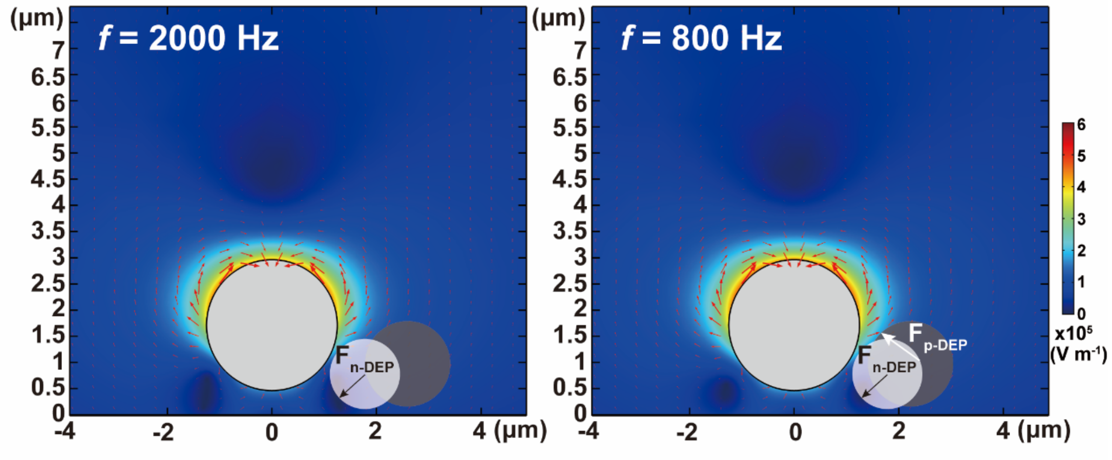

Supplementary Figure 8. Electric field strength distribution around a 2.5-μm dielectric sphere and its interactions with a P_1 patchy particle. The dielectric lobe of $\mathbf{P_1}$ particle is attracted at the low-field region while the metallic lobe feels repulsion from the central dielectric sphere when AC frequency f = 2,000 Hz. The reduced frequency (f = 800 Hz) leads to enhanced p-DEP attraction for metallic lobe resulting in a smaller bond angle. Red arrows represent the direction of electric field lines. Color bar: electric field strength.

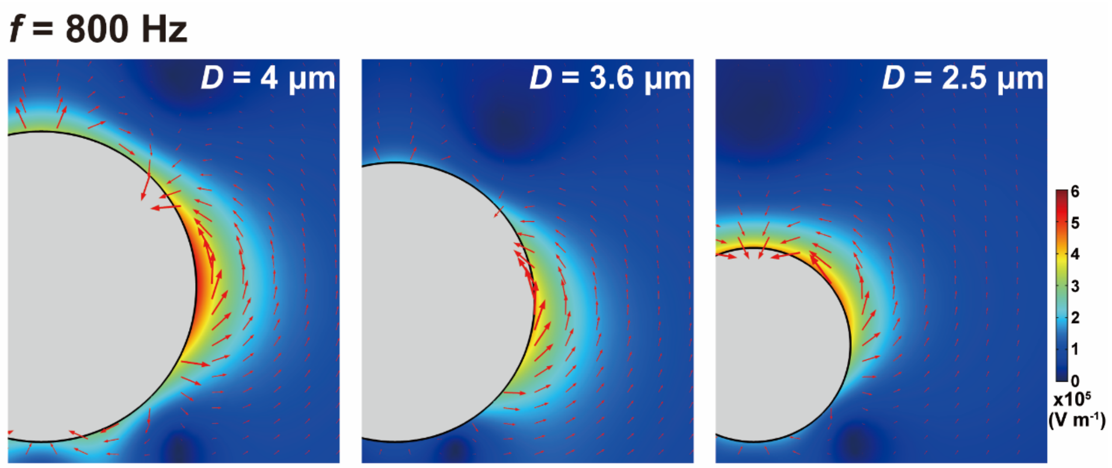

Supplementary Figure 9. Electric field distribution around dielectric spheres with different sizes. The nonuniform distribution of electric field at low AC frequencies can be also influenced by the size of dielectric sphere, in particular if the size is small. Such distribution is calculated and shown. The differences are due to the fact that particles with different sizes bear different effect from the electrode. For the smaller particles (D = 2.5 μm), more than half of the particle is directly polarized by charges on the electrode while for the biggest particle (D = 4 μm), only the lower part of the particle is directly affected. When the particle sizes are similar (e.g., 3.6 μm and 4 μm), their surrounding electric field distribution is similar. However, due to the same Debye length, the smaller particle has a thicker EDL relative to its size, which results in smaller impedance and less nonuniformity of local electric field. Red arrows represent the direction of electric field lines. Color bar: electric field strength.

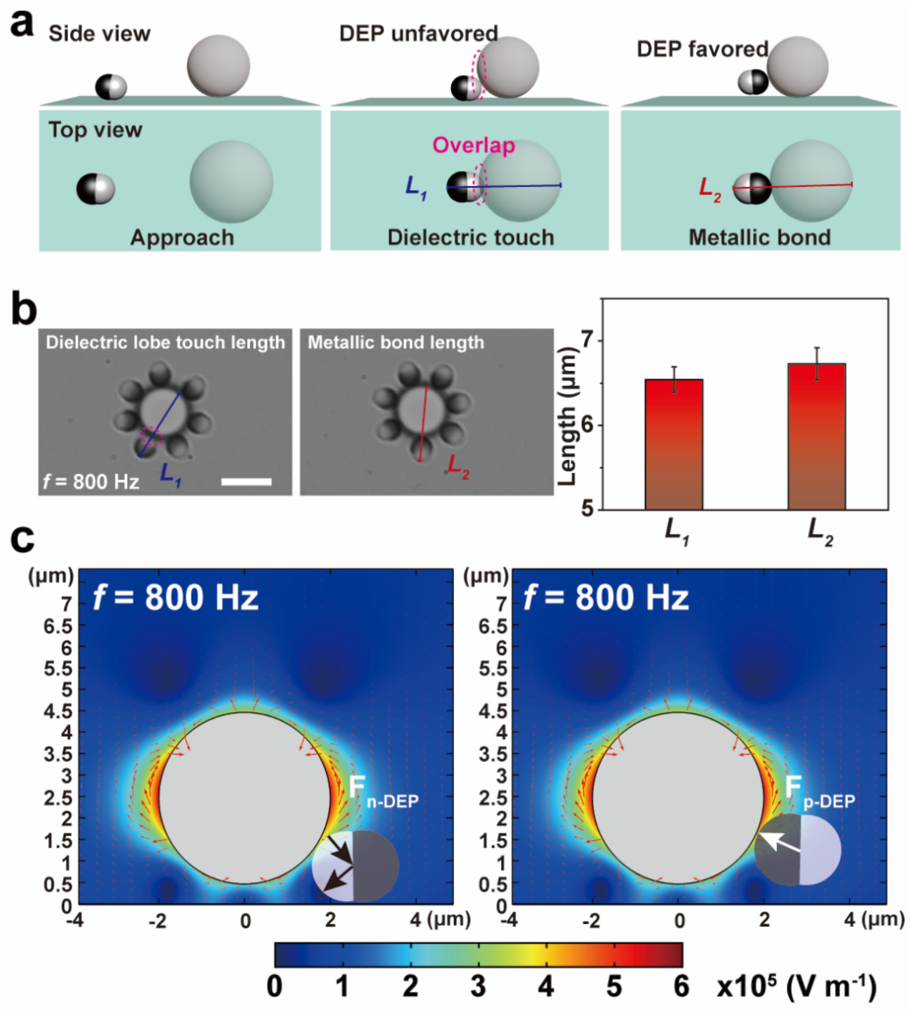

Supplementary Figure 10. Comparison between "dielectric touch" and metallic bond. a, The patchy particle $\mathbf{P_3}$ approaches and touches the bottom of the central sphere using its dielectric lobe, and then it turns, lifts slightly and finally forms the metallic bond. **b**, L_1 and L_2 are used to represent the dielectric touch length and the metallic bond length respectively. L_2 is longer than L_1 due to the overlap region of dielectric touch. This suggests that patchy particle is slightly lifted in the metallic bond configuration. The error bars refer to the standard deviation of lengths for multiple experiments. **c**, The dielectric lobe experiences both repulsive and attractive n-DEP force in the dielectric touch configuration, while, in the metallic bond configuration, the metallic lobe experiences the equatorial p-DEP attraction and avoid the bottom p-DEP repulsion because of the lift. The metallic bond is therefore energetically more favored considering the stronger polarization of metal. Red arrows represent the direction of electric field lines. Color bar: electric field strength. Scale bar: 4 μm.

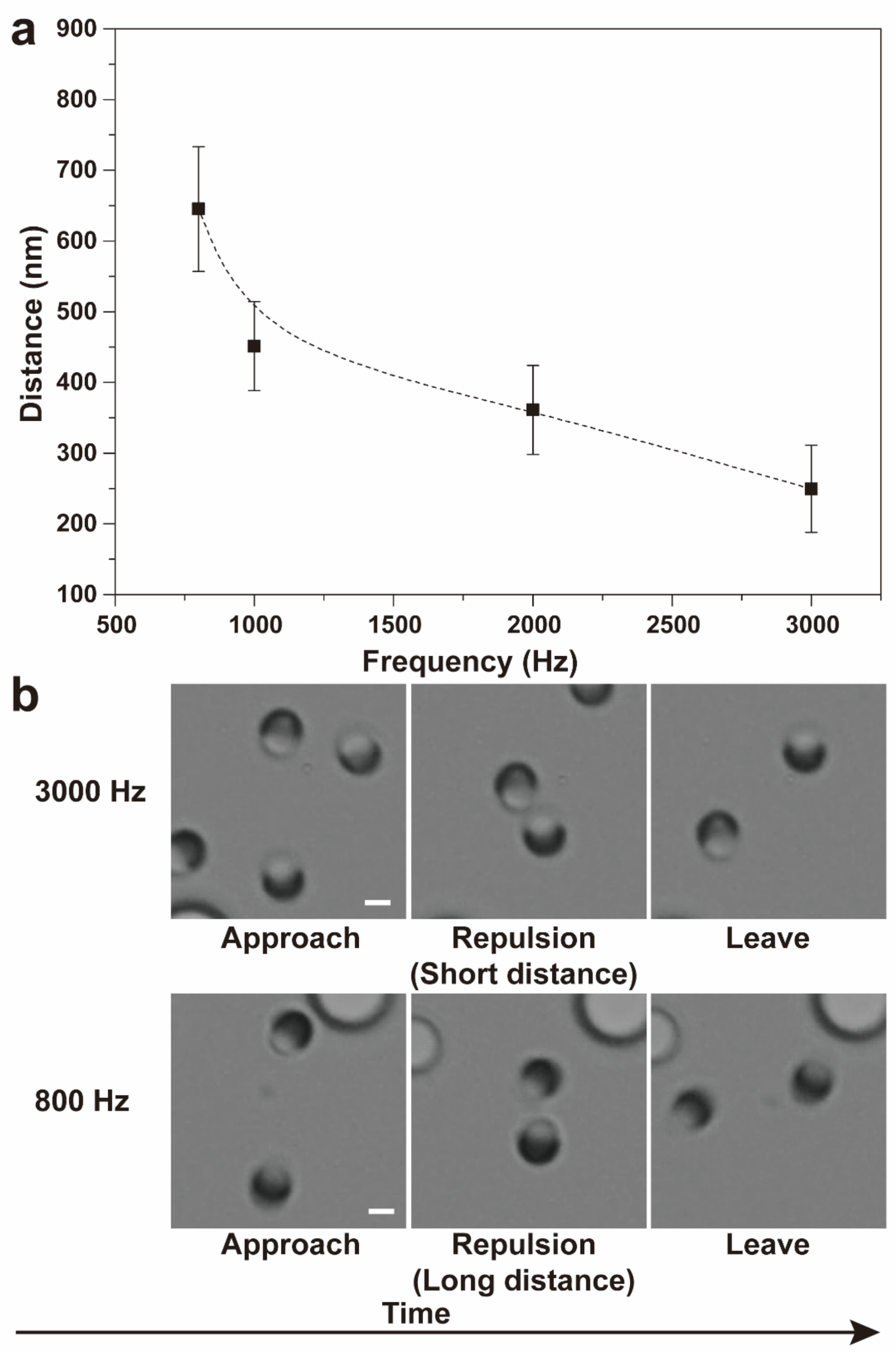

Supplementary Figure 11. Frequency-dependent repulsion between the dielectric lobes of two P_3 patchy particles. a, The minimum distance (black square) between the dielectric lobes of two patchy particles increases with reduced AC frequency. **b**, Optical microscope images demonstrate the minimum distances between the dielectric lobes when two particles approach each other at f = 3,000 and 800 Hz. See **Supplementary Movie 8** for more details. Scale bar: 1 μm. The error bars refer to the standard deviation of distances for multiple experiments.

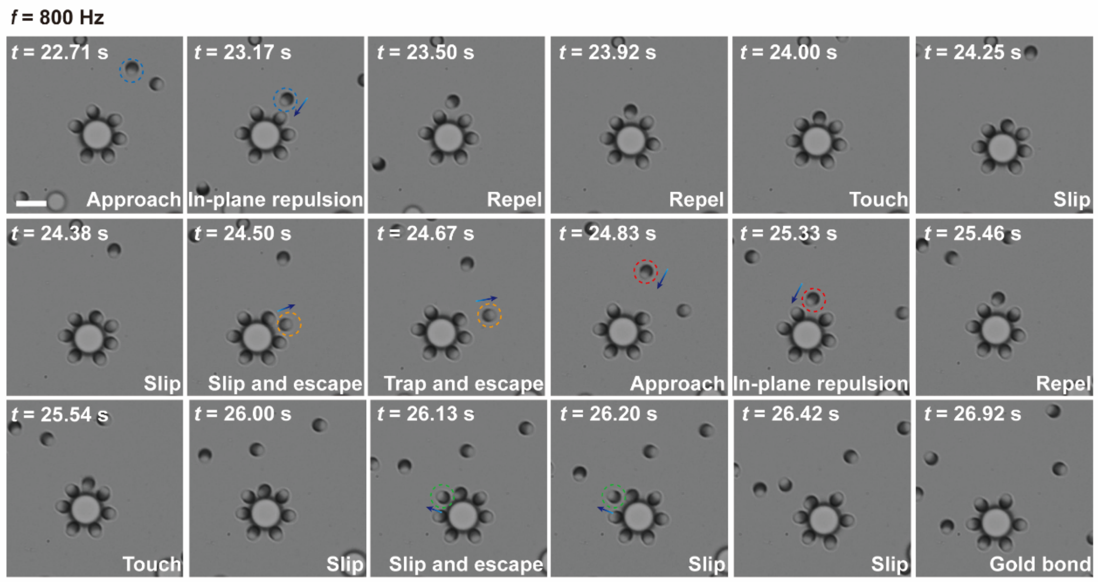

Supplementary Figure 12. Metallic bond substitution. The repulsion between the approaching and the bound patchy particles can cause the bound particle to escape, while a new bond can be established. Such process is shown. Scale bar: 4 µm.

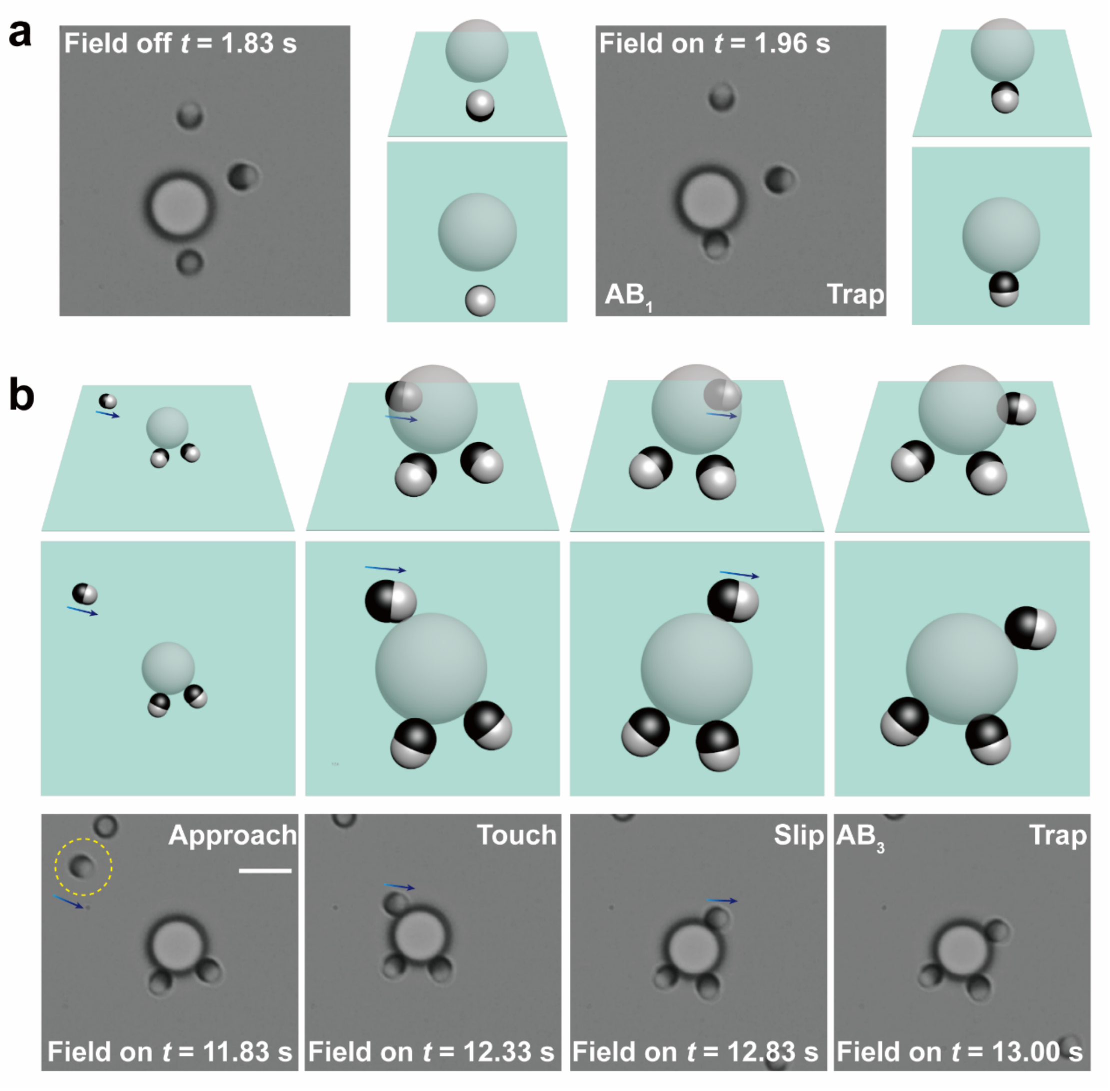

Supplementary Figure 13. Formation of AB_1 and AB_3 colloidal molecules with metallic bonds. a, The central particle traps the metallic lobes of nearby patchy particles. **b**, Similar to the binding process of AB_2, the formation of AB_3 colloidal molecule experiences the approach-touch-slip-turn-trap process. Scale bar: 4 μm.

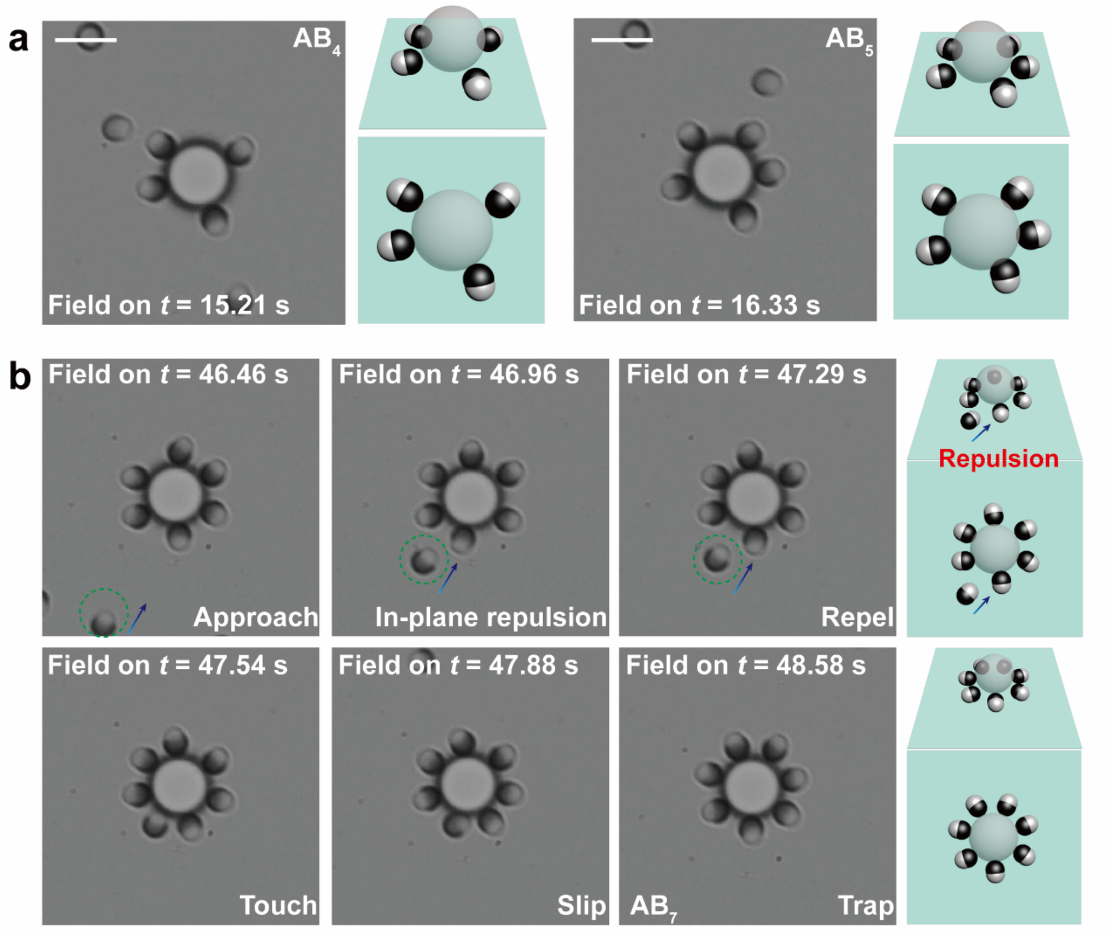

Supplementary Figure 14. Formation of AB_4, AB_5 and AB_7 colloidal molecules with metallic bonds. a, The colloidal molecules can grow to higher orders (AB_4 and AB_5) over time. **b**, Similar to the formation process of AB_6, the approaching patchy particle needs to overcome the repulsion from the bound ligand particles. Scale bar: 4 μm.

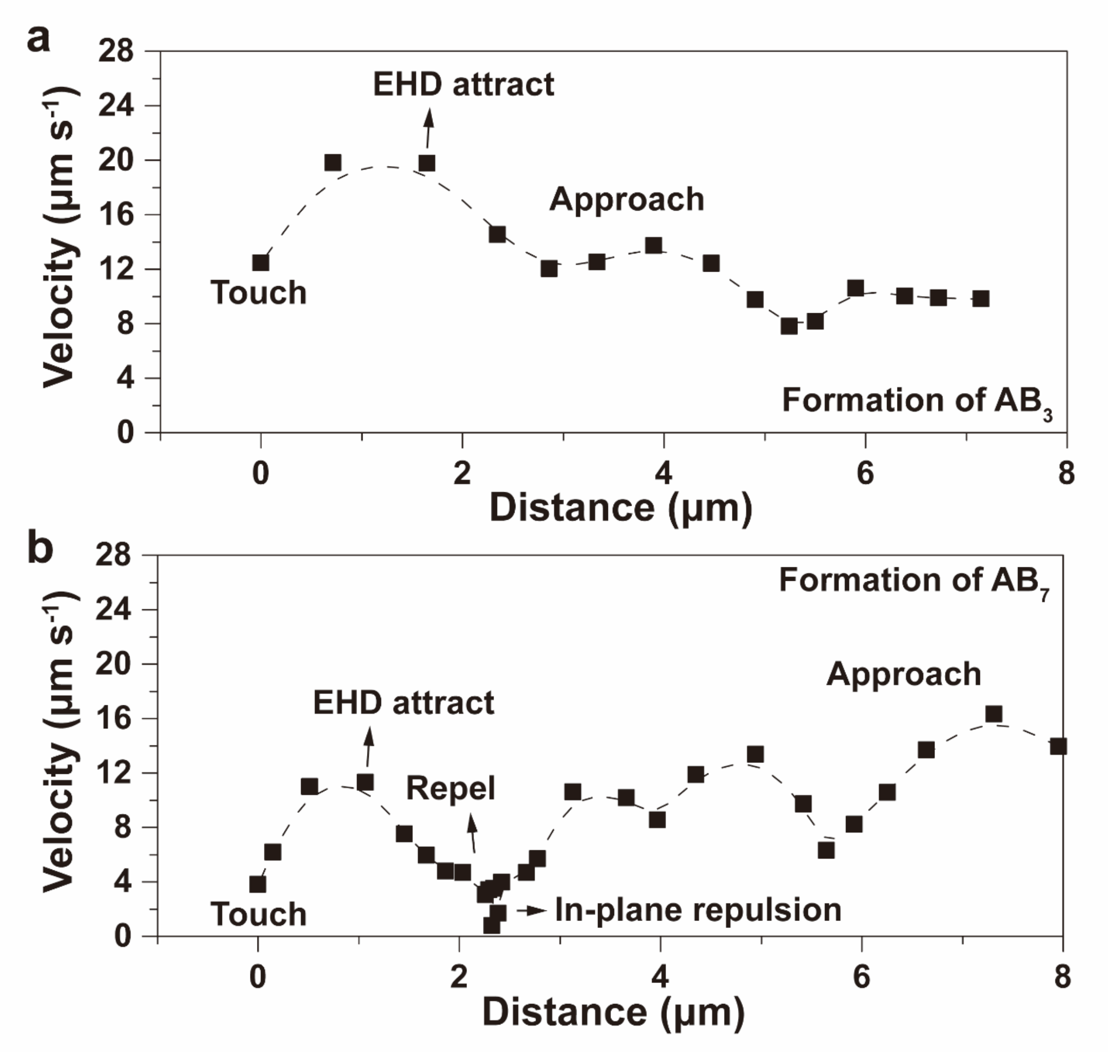

Supplementary Figure 15. The assembly kinetics of AB_3 and AB_7 show similar trend with the assembly of AB_2 and AB_6. a, For the low-order colloidal molecules (AB_n, $n \leq 4$), the addition of a ligand patchy particle is smooth. The patchy particle experiences an EHD acceleration about 2 μm away from the central particle and finally touches it. AB_3 is shown as an example. **b**, For the high-order colloidal molecules ($n \geq 5$), the formation of metallic bond undergoes a more complicated process and needs to overcome repulsion between the approaching and the bound patchy particles. The velocity of the approaching patchy particle decreases to almost 0 μm s^{-1} at around 2.5 μm away from the central sphere and repels the bound particles aside. Once enough room is available, the velocity of the approaching particle is accelerated again and finally forms the bond. AB_7 is shown as an example.

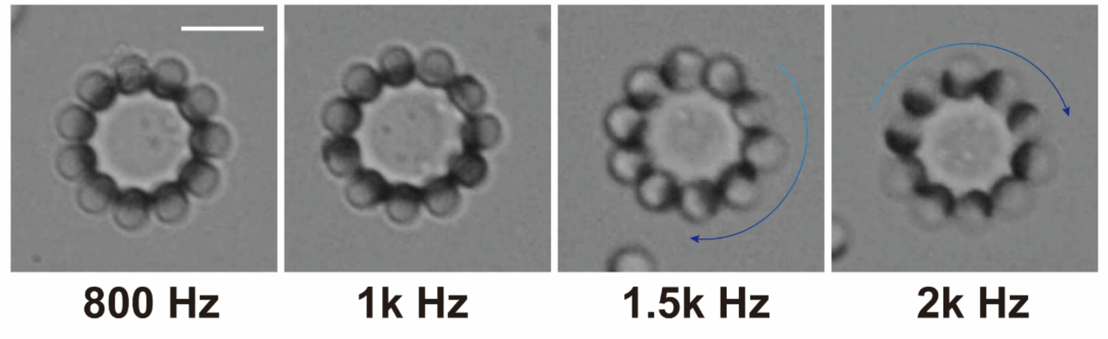

Supplementary Figure 16. AC frequency-dependent hybrid assembly. In the assembly of patchy particles with Yeast cells, the directionality of the formed colloidal bonds is weakened with increased AC frequency; the colloidal carousel structure can be observed at 1,500-2,000 Hz but not at lower frequencies. Scale bar: 4 μm.

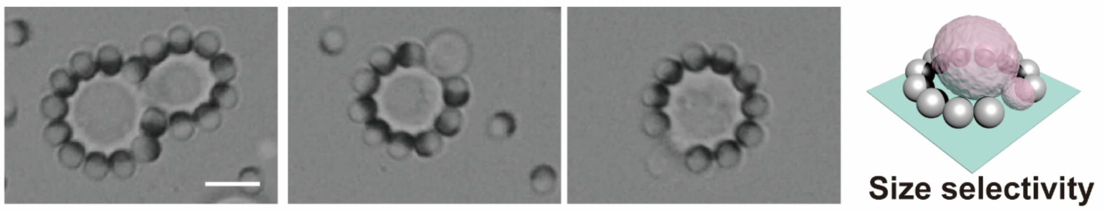

Supplementary Figure 17. Size selectivity of hybrid assembly. The patchy particles prefer to form metallic bonds to the big lobe of a dumbbell-shaped Yeast cells, demonstrating the size selectivity in the hybrid assembly. Scale bar: 4 μm.

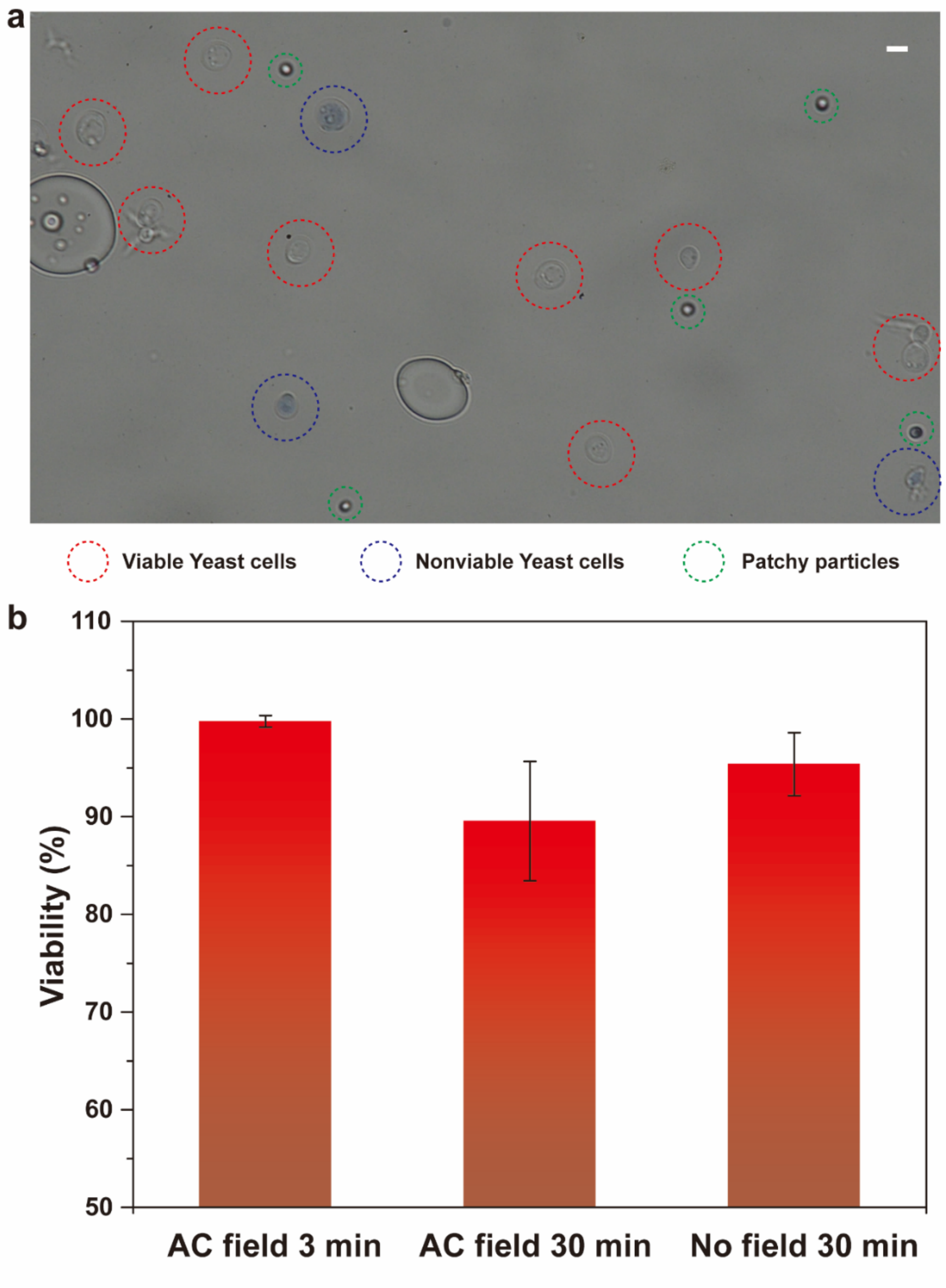

Supplementary Figure 18. The viability of Yeast cells. a, Optical microscope image of Yeast cells with patchy particles after 30 min of exposure under AC electric field for assembly (E = 2 × 10^5 V m^{-1}, f = 800 Hz). The dead cells showing blue tint is stained by Trypan blue (highlighted in blue circle), while the viable cells are colorless (highlighted in red circle). **b**, Viability of Yeast cells under different conditions: 3 min, 30 min as well as control set with no electric field applied but mixed with patchy particles for 30 mins. Scale bar: 4 µm. The error bars refer to the standard deviation of values for multiple experiments.

Supplementary Discussions.

Supplementary Discussion 1. Analysis of dielectrophoretic (DEP) forces.

For a spherical colloidal particle, the equation for calculating the DEP force can be expressed as:

$$F_{DEP} = 2\pi a^3 \varepsilon_0 \varepsilon_m Re[CM] \nabla |E|^2 \quad (1)$$

where a is the radius of the spherical particle, ε_0 is the absolute vacuum permittivity and ε_m is the relative permittivity of the media. $Re[CM]$ is the real part of the complex Clausius-Mossotti factor, which relies on both the frequency of applied field and the electrical properties of particle and media, representing the effective polarizability of a sphere in the media. Particle experiences a DEP force along the direction of $\nabla|E|^2$. In the frequency range we studied (800 to 2k Hz), the $Re[CM]$ almost remain constant; therefore, the variation of DEP force at different frequencies is mainly determined by the spatial distribution of field strength. To demonstrate the radial $\nabla|E|^2$ variation around the equator of a sphere, we record the field strength of one point on the equator (point A, Supplementary Figure 19) and another point (point B) 307 nm away from the point A along the radial direction in the finite element simulation. The corresponding $|E_A| - |E_B|$ at different frequency is plotted in Supplementary Figure 19 where the field strength difference between these two points are increased with the decrease of frequency. This proves that the DEP force will increase with the decrease of the frequency.

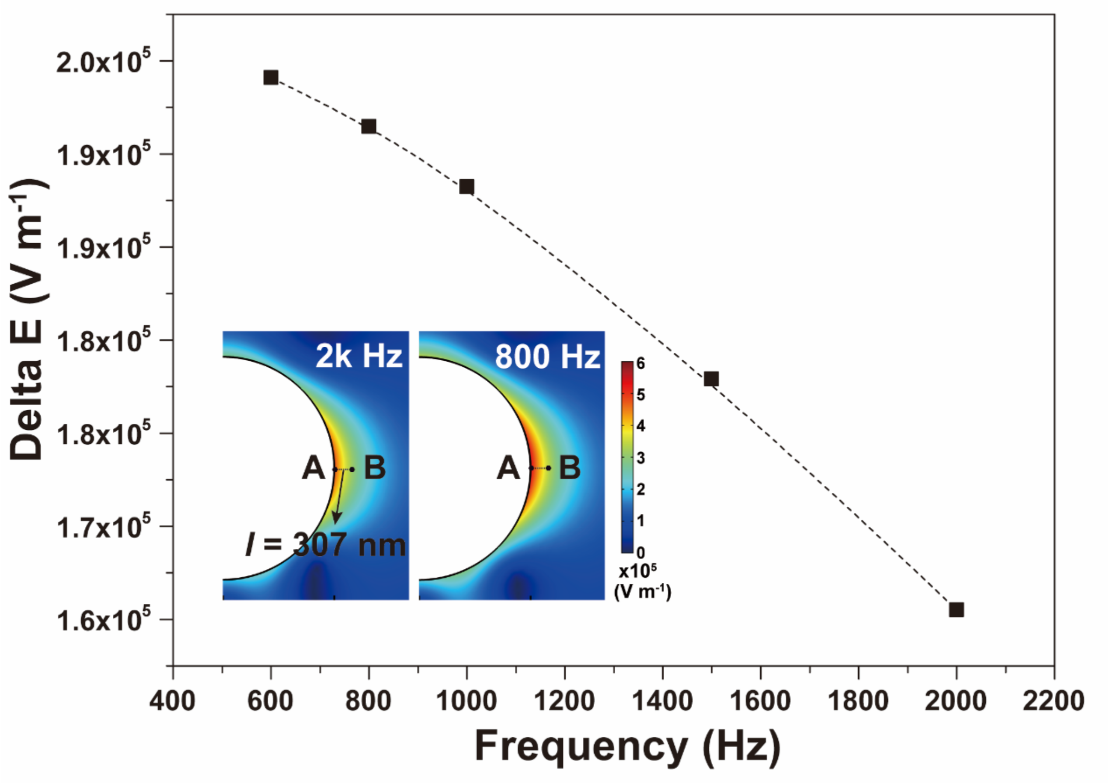

Supplementary Figure 19. Nonuniformity of local electric field strength generated around a dielectric sphere (4-μm) varies when the frequency of applied electric field changes. The difference of field strength between point A (on the equator of dielectric sphere) and point B as labeled (307 nm apart from A along the radial direction) decreases as the frequency of the electric field increases. Color bar: electric field strength.

Supplementary Discussion 2. Analysis of induced dipolar interaction.

The calculation of induced dipole force between two spherical colloidal particles A and B can be expressed as:

$$F_{dip} = 12\pi\varepsilon_0\varepsilon_m(R_A R_B)^3 C^2 E_{rms}^2 \left(\frac{1}{r_{AB}}\right)^4 [(3cos^2\gamma - 1)\hat{r} + (sin2\gamma)\hat{\gamma}] \quad (2)$$

Where R_A is the radius of the colloidal particles A, R_B is the radius of the colloidal particles B, r_{AB} is the distance between the two dipoles, E_{rms} is the root mean-squared magnitude of the applied electric field, ε_m is the relative permittivity of the media, ε_0 is the vacuum permittivity, and γ is the angle between the electric field and the connection of dipole centers. For a spherical colloidal particle, the polarizability C equals to the real part of the Clausius–Mossotti (CM) function such that $C=Re[K(\omega)]$. The $Re[K(\omega)]$ can be regarded as constant at the low frequency range (< 4k Hz). The attractive or repulsive dipolar force is determined by the angle

γ. Based on our experimental condition, the dielectric spheres should locate at the same plane above the bottom substrate, so the angle γ translates to the diameter ratio of two interacting particles. For example, if two particles are identical in size and in shoulder-to-shoulder connection, the angle γ equals to 90°, giving rise to maximum repulsion. While if one of the particles is small enough to fit the space between another big sphere and bottom substrate, this generates small angle γ and thus attractive dipolar interaction.

Supplementary Discussion 3. Effect of AC frequency on the dynamics of colloidal molecules possessing dielectric bonds.

We have shown in the main text that AC frequency is important in determining the final structures of the colloidal molecules such as the bond angles, which then translates to the dynamics of the assemblies. Besides, we note that multiple other factors in the system are frequency-dependent, such as the propulsion velocity of the constituent patchy particles and the EHD flow/force around the whole clusters, which collectively influences the final dynamic properties of the assemblies such as their steering curvatures, etc. Because both EHD and ICEP describe the effect of electric field on ionic charges induced by the field at fluid interfaces. In both cases, the generated electroosmotic flow U scales nonlinearly with the electric field, $U \propto E^2$. The flow depends in a complicated fashion on the frequency of the field, particle permittivity, and geometry (affect the direction of the flows when combined), etc. Therefore, we can further consider our results by superimposing the involved nonlinear electrokinetic mechanisms, EHD and ICEP, of two or multiple particles within an assembly. For an assembly of colloidal molecule, the total flow $U = U_{\mathrm{ICEP}} + U_{\mathrm{EHD}}$, where $U_{\mathrm{ICEP}} \propto E^2$ and $U_{\mathrm{EHD}} \propto E^2 f^{-1}$. Specifically, according to literatures[1], in the frequency range studied, for ICEP:

$$U_{ICEO} = \frac{9}{64}\frac{R}{1+\delta}\frac{\varepsilon\varepsilon_0 E^2}{\mu} \quad (3)$$

where R is the radius of sphere (sphere is adopted for simplicity), δ is the ratio of the differential capacitances of the compact and diffuse layers, ε is the relative permittivity of medium, ε_0 is vacuum dielectric constant, E is AC field strength, and μ is the viscosity of medium.

for EHD:

$$U_{EHDF} \approx b\,\frac{K''\kappa D}{f}\,\frac{\varepsilon\varepsilon_0 E^2}{\mu} \quad (4)$$

where K'' is the imaginary part of particles' polarization coefficient, κ is Debye screening length, D is the ion diffusivity in the medium, b is a prefactor that depends on the distance between the particle and the electrode, and f is AC frequency.

The velocity of patchy particle propulsion relies not only on the strength of the AC electric field, but also its frequency. It is noted that, as frequency decreases (e.g., from 2,000 Hz to 800 Hz), the propulsion velocity is increased.

Because the ICEP effect remains unchanged at the frequency range studied (800 to 2,000 Hz)[1-3], there must be another propulsion source. For the dielectric lobe of the patchy particle, there is an inward EHD flow, which is frequency dependent. Under identical condition, the metallic lobe will generate a reversed flow pattern[1], that is, an outward flow near the conducting substrate. This is due to the opposite sign of the imaginary part of the materials polarization coefficient. The net EHD flow of these two lobes thus propel the patchy particle to the same direction as ICEP (Supplementary Figure 20a), thus enhancing the overall propulsion when reducing the field frequency (Supplementary Figure 20d). This can be confirmed by the velocity change of the patchy particles.

The propulsion force of patchy particles can be calculated using the Stoke's equation:

$$F_{propulsion} = 6\pi\eta R v \quad (5)$$

where η is the viscosity of the medium, R is the radius of the particle and v is the velocity of the patchy particles. According to the equation, the magnitude of propulsion force is linearly proportional to the velocity of patchy particles.

We notice that at the high frequency (f = 2k Hz), where the EHD flow is weak, the shape of patchy particle also influences on the swimming velocity due to the different exposure of metallic lobes. However, at the low frequency (f = 800 Hz), the EHD flow dominates the propulsion and narrows down the velocity difference due to small changes in shape (Supplementary Figure 20d).

The colloidal molecules formed by dielectric bond can swim cooperatively when the number of ligands $n \geqslant 2$ (Supplementary Figure 20c). We investigate propulsion velocity of colloidal molecules with $n = 2$ (AB_2) and $n = 3$ (AB_3). In both cases, the velocity increases with

decreased field frequency (Supplementary Figure 20e).

As shown in Supplementary Figure 20f, the steering radius (we track the central of mass of big sphere) is determined by the inward component of the propulsion force ($F_{propulsion} \cdot \sin\theta$), which is predetermined by both the magnitude of force and the bond angle θ. Since both the propulsion force of patchy particles and bond angle are influenced by the applied field frequency, the final steering radius in Figure 3f is the result reflecting these two factors.

In colloidal molecules assumed by metallic bonds, because the direction of ICEP and EHD is opposite, the relevant flows are balanced (Supplementary Figure 20b). As a result, the colloidal molecules stayed rather static. Also, at low frequency, the large EHD flow from the central sphere may also contribute to the formation of the bond.

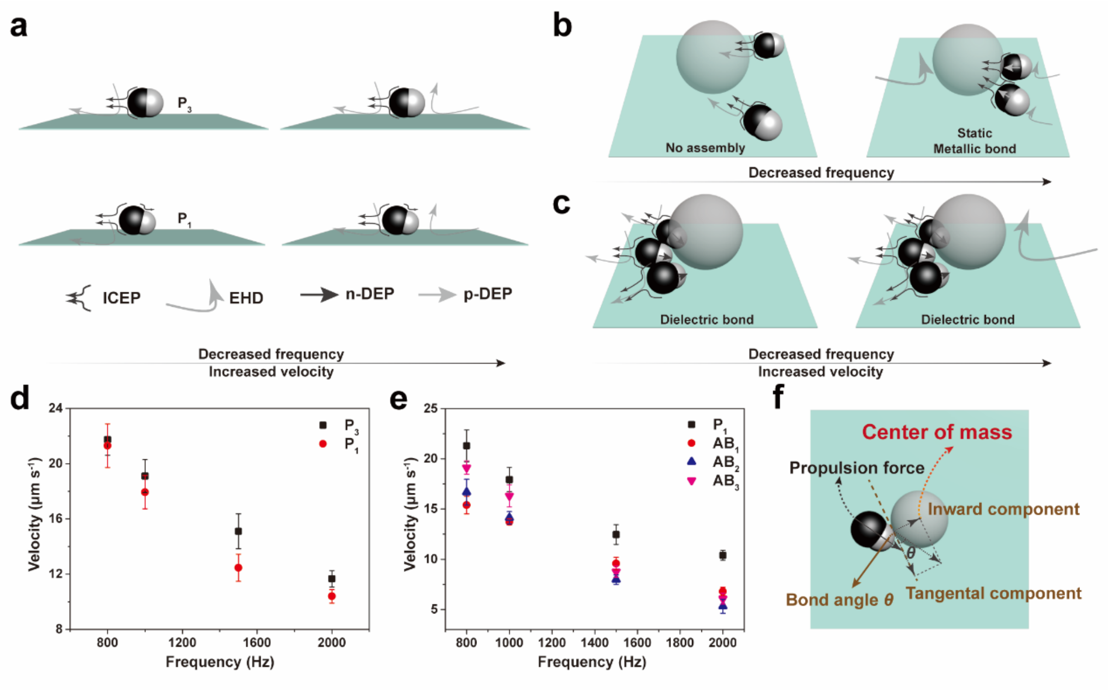

Supplementary Figure 20. Frequency dependence of particle propulsion. a, The velocity of propulsion for patchy particles (**P_1** and **P_3**) increases at low frequency due to EHD enhancement. The strong EHD flow at low *f* also influences the dynamics of colloidal molecules with metallic bonds (**b**) and dielectric bonds (**c**). For molecules with dielectric bonds, the propulsion velocity is increased; for molecules with metallic bonds, the propulsion is halted. Plots showing the propulsion velocity of **P_1** and **P_3** (**d**) and colloidal molecules with dielectric

bonds (**e**) as a function of f in DI water ($E = 2 \times 10^5$ V m^{-1}). The error bars refer to the standard deviation of velocities for multiple experiments. **f**, Force analysis of the steering of AB_1 molecules with a dielectric bond.

Supplementary Discussion 4. The difference between synthetic particles and biological "particles" (living cells).

Living cells are in many ways different from synthetic (polymeric) particles and show interesting properties in the assembly of colloidal molecules.

First, the permittivity of living cells is around ε_{cell} = 30[4], much smaller than that of polymeric particles ($\varepsilon_{polymer}$ = 3). This means the dielectric contrast between the cell and the media (DI water, ε_{DI}=80) is smaller. Because the dielectric contrast is the key for generating the local nonuniform electric field, the decreased contrast can reduce the nonuniformity of the field and leads to weakened DEP force.

Second, the zeta potential of cells (for Yeast cell, $\boldsymbol{\zeta}$=-20~30 mV)[5] is smaller than synthetic sphere we used ($\boldsymbol{\zeta}$ = -50 mV); therefore, the impedance of the EDL capacitor is weakened and the frequency dependence DEP force becomes less apparent.

The weakened DEP force gives rise to colloidal bonds (both dielectric and metallic) that are not as strong. For AB_1 colloidal molecules of yeast cell and patchy particle $\mathbf{P_2}$ (dielectric bond), the bound patchy particle seems to only slide around the rim of cell, the assembly showing little steering as the cell mostly stays static. In addition, the number of bonds per cell as well as over the whole sample is small indicative of weak bonds. In the case of metallic bonds, pure metallic bonds are only observed when AC frequency is low, f < 1,000 Hz. At higher frequencies, a mixed bond configuration is often observed. One of the striking examples is the formation of the colloidal carousel at f = 1,500-2,000 Hz.

Third, the shape of living cells is irregular and may deform upon forces. Cells are not perfectly spheres (or rods); the surface can possess some degrees of roughness. This could cause heterogeneity of the spatial distribution of induced electric field around the cell, and eventually influence the selectivity and directionality of colloidal bonds. With various force exerted when ligand particles are attached, cells may change their shape slightly to accommodate more ligand particles. Examples are the colloidal molecules with really tightly packed patchy particles.

Finally, the living cells are composed of many distinct parts with distinct substances. The materials distribution may affect their electrical properties. We note that the situation is more complicated as electroporation may take place when cells are under electric field; this may cause cytoplasm to diffuse through cytomembrane and thus change the permittivity and conductivity of the cell and the surrounding media.

Supplementary References

1 Wang, S., Ma, F., Zhao, H. & Wu, N. Bulk synthesis of metal-organic hybrid dimers and their propulsion under electric fields. *ACS Appl. Mater. Interfaces* **6**, 4560-4569, (2014).

2 Shields, C. W. *et al.* Supercolloidal Spinners: Complex Active Particles for Electrically Powered and Switchable Rotation. *Adv. Funct. Mater.* **28**, 1803465, (2018).

3 Gangwal, S., Cayre, O. J., Bazant, M. Z. & Velev, O. D. Induced-charge electrophoresis of metallodielectric particles. *Phys. Rev. Lett.* **100**, 058302, (2008).

4 Yao, J., Kodera, T., Sapkota, A., Obara, H. & Takei, M. Experimental study on dielectric properties of yeast cells in micro channel by impedance spectroscopy. 25th 2014 International Symposium on Micro-NanoMechatronics and Human Science (MHS). *Institute of Electrical and Electronics Engineers (IEEE),* Nagoya, Japan, 2014, pp. 1-4.

5 Thonart, P., Custinne, M. & Paquot, M. Zeta potential of yeast cells: application in cell immobilization. *Enzyme Microb. Technol.* **4**, 191-194, (1982).
