# Supplementary material for: Active colloidal molecules assembled via selective and directional bonds
Source: Nat Commun. 2020 May 29;11:2670. doi: 10.1038/s41467-020-16506-z (PMC7260206; doi:10.1038/s41467-020-16506-z)
Supplement: Supplementary file 3 — Description of Additional Supplementary Files [file 41467_2020_16506_MOESM3_ESM.pdf]

Description of Additional Supplementary Files

File Name: Supplementary Movie 1
Description: Colloidal molecules are assembled by dielectric spheres 4 and 1 μm in diameter (D); they propel under AC electric field (frequency f = 600 Hz). The movie was captured and played at 24 fps.

File Name: Supplementary Movie 2
Description: Size selectivity for the formation of colloidal molecules are verified by mixing big (D = 4 μm), medium (D = 2.5 μm) and small (D = 1 μm) dielectric spheres under AC electric field (frequency f = 600 Hz). The 1-μm small sphere selectively binds to the 4-μm big sphere, while both small-medium and medium-big spheres show obvious repulsion and no assembly. The movie was captured and played at 24 fps.

File Name: Supplementary Movie 3
Description: Loose and dynamic bond is observed by the binary assembly of 4-μm and 1.5-μm dielectric spheres under AC electric field (f = 2,000 Hz). The assemblies oscillate between bonding and un-bonding frequently. The amplitude of oscillation becomes smaller when decreasing the AC frequency to f = 600 Hz. The movie was captured and played at 24 fps.

File Name: Supplementary Movie 4
Description: Tracer particles (PS spheres) surrounding a 4-μm dielectric sphere form static bonds with the big sphere close to the substrate, but are ejected away at the top of the big sphere. This indicates an inward (with respect to the 4-μm sphere) EHD flow near the substrate (f = 600 Hz). The movie was captured and played at 24 fps.

File Name: Supplementary Movie 5
Description: High-order colloidal molecules (AB5) constantly reconfigure as the ligand particles slide along the rim of central sphere, which leads to the simultaneous velocity change (f = 600 Hz). The movie was captured and played at 24 fps.

File Name: Supplementary Movie 6
Description: Colloidal molecules with "dielectric bonds" are formed by metallodielectric patchy particles and central dielectric spheres. We show that the bond angle and propulsion trajectories of the resulting colloidal molecules can be tuned by either the particle size or the AC frequency. The movie was captured and played at 24 fps.

File Name: Supplementary Movie 7
Description: Formation of colloidal molecules with "metallic bond" by metallodielectric patchy particles and central dielectric spheres. We show that the metallic bond is sensitive to the size of central dielectric spheres and AC frequency. The movie was captured and played at 24 fps.

File Name: Supplementary Movie 8
Description: Small distance between two the dielectric lobes of two P3 particles are observed at f= 3,000 Hz indicating small dipolar repulsion, while the distance is increased

dramatically at AC frequency f = 800 Hz suggesting strong dipolar repulsions. The movie was captured and played at 24 fps.

File Name: Supplementary Movie 9
Description: Under AC electric field (f = 800 Hz), PIV and PV bind both their metallic and dielectric lobes to the central sphere (D = 4 μm), forming the "mixed bond" due to weakened bond directionality, while PVII and PVIII show highly directional metallic bond due to their larger dielectric lobes. The movie was captured and played at 24 fps.

File Name: Supplementary Movie 10
Description: The E. Coli cells lie down and form self-propelled hybrid colloidal molecules at low frequency (f = 300 Hz). The E. Coli cells stand up and the increased repulsion inhibits the assembly at high frequency (f = 1,000 Hz). The movie was captured and played at 24 fps.

File Name: Supplementary Movie 11
Description: The Yeast cells assemble with small dielectric sphere generating self-propelled hybrid colloidal molecules under AC electric field (f = 2,000 Hz). The movie was captured and played at 24 fps.

File Name: Supplementary Movie 12
Description: The Yeast cells and E. Coli assemble to form AB-type colloidal molecules AC electric field (f = 300 Hz) that propel. The movie was captured and played at 24 fps.

File Name: Supplementary Movie 13
Description: The metallodielectric patchy particle P1 bind to Yeast cell with dielectric bond forming steering hybrid colloidal molecules under AC electric field (f = 1,500 Hz). The movie was captured and played at 24 fps.

File Name: Supplementary Movie 14
Description: Formation of colloidal molecules with metallic bond by patchy particle P4 and Yeast cell under AC electric field (f = 800 Hz). The movie was captured and played at 24 fps.

File Name: Supplementary Movie 15
Description: At a higher AC frequency, all the patchy particles slide to one side of the Yeast cell. When the surrounding of Yeast cell is saturated by P4, the patchy particles synchronize and start to propel around the Yeast cell giving rise to a "Colloidal Carousel" structure only if most patchy particles align their direction. This is a stochastic process; otherwise, patchy particles get stuck leading to a static assembly. The movie was captured and played at 24 fps.
